# Comparative Evaluation of Anti-microbial Activity of Herbal, Homeopathic, and Conventional Dentifrices Against Oral Microflora Using the Disc Diffusion Method: An In Vitro Study

**DOI:** 10.7759/cureus.62197

**Published:** 2024-06-11

**Authors:** Manoj K, Dharani S, Shakthivel P, Divyasharan S, Shalini B, Nandhinidevi G, Ritu Agarwal, Gayathri G, Sobana R

**Affiliations:** 1 Pediatric Dentistry, Priyadarshini Dental College and Hospital, Thiruvallur, IND; 2 General Dentistry, Shrivatsa Dental Clinic, Sholingur, IND; 3 General Dentistry, V Dental Care, Chennai, IND; 4 General Dentistry, Ivory Dentistry, Salem, IND; 5 Pediatric Dentistry, Sri Ramachandra Dental College & Hospital, Chennai, IND; 6 Pediatric Dentistry, Ivory Dentistry, Salem, IND; 7 Dentistry, Paedodontics and Preventive Dentistry, Awadh Dental College and Hospital, Jamshedpur, IND

**Keywords:** oral microflora, homeopathy, herbal, dentifrice, anti-microbial

## Abstract

Aim

To assess the antimicrobial activity of herbal, homeopathic, and conventional dentifrices against oral microorganisms.

Methodology

Mueller Hilton agar was used to cultivate distinct strains of *Streptococcus mutans* and *Enterococcus faecalis*, whereas *Candida albicans* was cultured on a potato dextrose agar medium. Diffusion ratios of 1:5, 1:10, and 1:15 were obtained by diluting 1 gram of each dentifrice (KP Namboodiri, Homeodent, and* *Colgate Strong Teeth) in 4 ml, 9 ml, and 14 ml of distilled water, respectively. The culture medium was filled with sterile discs. Twenty μl of each dilution of prepared dentifrice formulations were incorporated using a micropipette. The agar plates were incubated for 24 hours at 37ºC.

Result

The findings indicate that there was a higher zone of inhibition against *Streptococcus mutans *with herbal dentifrice at 10 mm, 8 mm, and 6.5 mm, followed by conventional dentifrice at 10 mm, 7.5 mm, and 7 mm, and the lowest with homeopathic dentifrice at 8 mm, 7 mm, and 7 mm at 1:5, 1:10 and 1:15 dilutions, respectively. Conventional dentifrice was found to inhibit *Enterococcus faecalis *at 9 mm, 8 mm, and 7 mm with 1:5, 1:10, and 1:15 dilutions followed by herbal dentifrice at 9 mm, 7 mm with 1:5, 1:10 dilutions, and no inhibition at 1:15 dilution. In contrast, homeopathic dentifrice displayed no inhibition at 1:5, 1:10, and 1:15 dilutions. Neither homeopathic nor conventional dentifrices inhibited *Candida albicans*, but herbal dentifrices showed a 10 mm zone of inhibition at 1:10 dilution.

Conclusion

Conventional and herbal dentifrices were found to be more effective against *Streptococcus mutans *than the homeopathic dentifrice used in the study, whereas herbal dentifrice was more effective against* Candida albicans *when compared to conventional and homeopathic dentifrices.

## Introduction

Dental plaque accumulation on teeth is a problem from both a pathological and a cosmetic point of view. Dental caries, gingivitis, periodontal issues, halitosis, and numerous other oral health issues are all caused by the presence of plaque. Plaque can be removed or controlled mechanically using a variety of tools, such as toothbrushes and dental floss [1].

The most widely established technique for managing plaque and gingivitis is through mechanical means. However, most people's mechanical plaque control is insufficient. Therefore, in addition to regular mechanical oral hygiene regimes, they also need to utilize chemical control methods such as using an anti-microbial dentifrice [2].

Using a dentifrice in conjunction with a toothbrush, one can prevent periodontal and caries problems by eliminating debris, material particles, and bacterial plaque [3]. Due to its anti-inflammatory, antibacterial, analgesic, and antioxidant properties, natural dentifrice has become increasingly popular lately. Homeopathy uses a material in smaller dosages with no negative side effects to treat the disease symptoms [4]. Additives, including humectants, binders, detergents, flavors, and preservatives, are found in conventional dentifrices [5]. Because of their proven ability to effectively combat bacteria that cause dental caries, their antibacterial qualities make them a perfect ingredient for toothpaste. Using these agents or components in toothpaste also contributes to the management and reduction of gingivitis and plaque [4].

Several toothpaste brands on the market aim to fight oral microorganisms. Thus, this study was conducted using the disc diffusion method to examine the antimicrobial efficiency of herbal, homeopathic, and conventional dentifrices.

## Materials and methods

Study setting

This in vitro study was carried out at the Priyadarshini Dental College and Hospital, Pandur, Tamilnadu, India, during the period between 05-02-2024 and 10-02-2024.

Microorganisms

Three strains of microorganisms, namely, *Streptococcus mutans, Enterococcus faecalis*, and *Candida albicans*, were used in the current in vitro study.

On Mueller Hilton agar media, *Streptococcus mutans* and *Enterococcus faecalis *were cultured while *Candida albicans* was cultured on a potato dextrose agar medium.

Dentifrice formulations

Three commercially available dentifrices were used in the study, namely, herbal dentifrice - KP Namboodiri (KP Namboodiri's Ayurvedics, Kerala, India), homeopathic dentifrice - Homeodent (SBL Pvt. Ltd., New Delhi, India), and conventional fluoridated dentifrice - Colgate Strong Teeth (Colgate-Palmolive Company, India).

*Ingredients* *of KP Namboodiri*

KP Namboodiri contains extracts of ginger, black pepper, long pepper, *Terminalia chebula*, gooseberry, licorice, camphor, menthol, and clove oil, along with calcium carbonate, glycerine, di-calcium phosphate dihydrate, sodium lauryl phosphate, sodium carboxymethyl cellulose, red ochre, and sodium benzoate.

Ingredients of Homeodent

Ingredients of Homeodent include *Calendula officinalis*, *Hamamelis virginica*, *Plantago major* seed oil, calcium carbonate, sodium fluoride, and sorbitol.

Ingredients of Colgate Strong Teeth

Colgate contains ingredients such as calcium carbonate, sorbitol, sodium lauryl sulfate, silica, titanium dioxide, sodium silicate, sodium monofluorophosphate, sodium bicarbonate, potassium nitrate, benzyl alcohol, sodium saccharin, and limonene.

**Figure 1:**
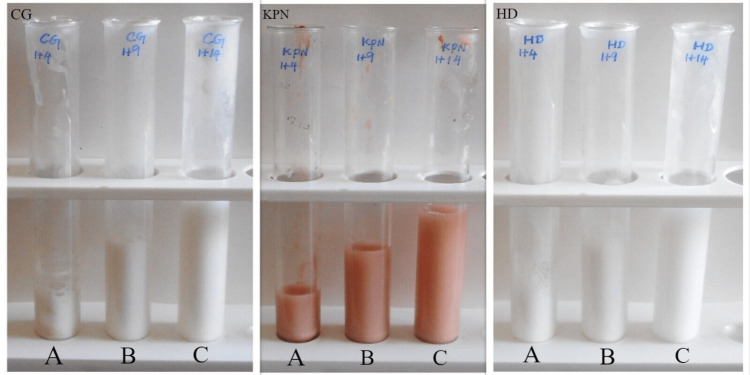
Dentifrice formulations at three different dilutions CG – Colgate Dentifrice; KPN - KP Namboodiri Dentifrice; HD - Homeodent Dentifrice A - 1:5 dilution; B - 1:10 dilution; C - 1:15 dilution

Diffusion ratios of 1:5, 1:10, and 1:15 were obtained by diluting 1 g of each selected dentifrice in 4 ml, 9 ml, and 14 ml of distilled water, respectively (Figure 1).

Agar disc diffusion method

Preparation of Bacterial Inoculum

Stock cultures were maintained on nutrient agar slants at 4 °C. To prepare active cultures for research, a loop full of cells from the stock cultures was transferred to test tubes containing nutrient broth for bacteria, which were then cultured for 24 hours at 37 ºC. The agar disc diffusion method was used to carry out the assay.

Antibacterial Activity

Using the Muller Hinton Agar (MHA) medium and the disc diffusion method, the antibacterial activity of the sample was assessed. MHA contains beef extract, acid hydrolysate of casein, starch, and agar, and is a non-selective, non-differential medium. One gram of agar was added after the 3.8 grams of MHA medium had been dissolved in 100 milliliters of distilled water. The medium was then stored in order to be sterilized. Sterilized petri plates were filled with the media, which was then left to harden for an hour. Using a sterile swab moistened with the bacterial suspension, the inoculums were applied to the solid plates once the medium had hardened. Discs were prepared with 20 µl of samples (KP Namboodiri, Homeodent, and Colgate Strong Teeth toothpaste) of three different concentrations (1+4, 1+9, and 1+14), which were subsequently placed on MHA plates. These plates were incubated at 37 ºC for 24 hours. Next, the diameter of the zone of inhibition was measured to estimate the microbial growth.

Preparation of Fungal Inoculum

Stock cultures were maintained on potato dextrose agar slants at 4 °C. In order to prepare active cultures for research, a loop full of cells from the stock cultures was transferred to test tubes containing nutrient broth for bacteria, which were then cultured for 24 hours at 37 ºC. The agar disc diffusion method was used to carry out the assay.

Antifungal Activity

Using the potato dextrose agar (PDA) medium and the disc diffusion method, the antifungal activity of the sample was assessed. PDA is a common microbiological basal growth media made from a potato infusion and dextrose for the growth of the fungi. One gram of agar was added after the 4.4 grams of potato dextrose agar medium had been dissolved in 100 milliliters of distilled water. The medium was then stored in order to be sterilized. Sterilized petri plates were filled with the media, which was then left to harden for an hour. Using a sterile swab moistened with the fungal suspension, the inoculums were applied to the solid plates once the medium had hardened. Discs were prepared with 20 µl of samples (KP Namboodiri, Homeodent, and Colgate Strong Teeth toothpaste) of three different concentrations (1+4, 1+9, and 1+14), which were subsequently placed on PDA plates. These plates were incubated at 37 ºC for 24 hours. Next, the diameter of the zone of inhibition was measured to estimate the microbial growth.

## Results

**Figure 2:**
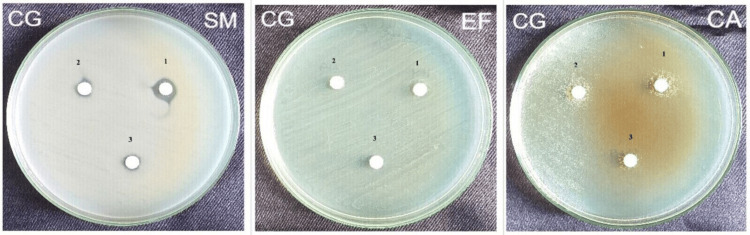
Zone of inhibition produced by Conventional dentifrice at three different dilutions against Streptococcus mutans, Enterococcus faecalis, and Candida albicans CG - Colgate Dentifrice; SM - Streptococcus mutans; EF - Enterococcus faecalis; CA - Candida albicans 1 - 1:5 Dilution; 2 - 1:10 Dilution; 3 - 1: 15 Dilution

The zone of inhibition produced by conventional dentifrice (CG) at 1:5 dilution against *Streptococcus mutans *and *Enterococcus faecalis* was 10 mm and 9 mm, respectively, whereas at 1:10 dilution against *Streptococcus mutans *and *Enterococcus faecalis, *it was 7.5 mm and 8 mm. And the zone of inhibition produced at 1:15 dilution against *Streptococcus mutans *and *Enterococcus faecalis* was 7 mm and 7 mm. There was no zone of inhibition against *Candida albicans* at all three dilutions (Figure 2).

**Figure 3:**
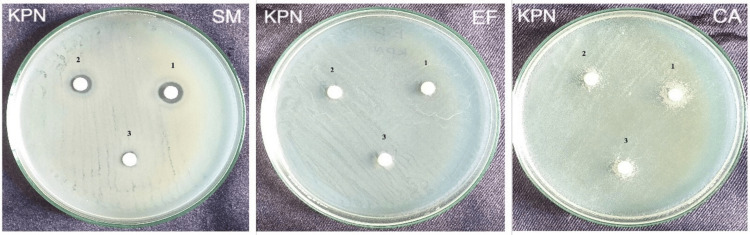
Zone of inhibition produced by Herbal dentifrice at three different dilutions against Streptococcus mutans, Enterococcus faecalis, and Candida albicans KPN - KP Namboodiri Dentifrice; SM - Streptococcus mutans; EF - Enterococcus faecalis; CA - Candida albicans 1 - 1:5 Dilution; 2 - 1:10 Dilution; 3 - 1: 15 Dilution

The zone of inhibition produced by herbal dentifrice (KPN) at 1:5 dilution against *Streptococcus mutans* and *Enterococcus faecalis *was 10 mm and 9 mm, respectively, whereas, at 1:10 dilution against *Streptococcus mutans, Enterococcus faecalis, and Candida albicans, *it was 7.5 mm, 8 mm, and 10 mm, respectively. The zone of inhibition produced at 1:15 dilution against *Streptococcus mutans* was 6.5 mm. There was no zone of inhibition against *Enterococcus faecalis* at 1:15 dilution and *Candida albicans* at 1:5 and 1:15 dilutions (Figure 3).

**Figure 4:**
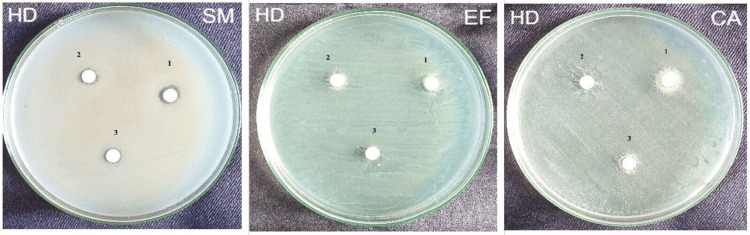
The zone of inhibition produced by homeopathic dentifrice at three different dilutions against Streptococcus mutans, Enterococcus faecalis, and Candida albicans HD - Homeodent Dentifrice; SM - Streptococcus mutans; EF - Enterococcus faecalis; CA - Candida albicans 1 - 1:5 Dilution; 2 - 1:10 Dilution; 3 - 1:15 Dilution

The zone of inhibition produced by homeopathic dentifrice (HD) at 1:5 dilution against *Streptococcus mutans* was 8 mm, whereas at 1:10 dilution against *Streptococcus mutans* was 7 mm. The zone of inhibition produced at 1:15 dilution against *Streptococcus mutans *was 7 mm. There was no zone of inhibition against *Enterococcus faecalis *and *Candida albicans* at all three dilutions (Figure 4).

Herbal dentifrice at 10 mm, 8 mm, and 6.5 mm had a higher zone of inhibition against *Streptococcus mutans* than conventional dentifrice at 10 mm, 7.5 mm, and 7 mm. Homeopathic dentifrice at 8 mm, 7 mm, and 7 mm at 1:5, 1:10, and 1:15 dilutions had the least zone of inhibition. *Enterococcus faecalis* was inhibited by conventional dentifrice at 9 mm, 8 mm, and 7 mm at 1:5, 1:10, and 1:15 dilutions. Herbal dentifrice was found to exhibit no inhibition at 1:15 dilution and exhibited inhibition at 9 mm and 7 mm with 1:5 and 1:10 dilutions. Homeopathic dentifrice, on the other hand, showed no inhibition at dilutions of 1:5, 1:10, and 1:15. Homeopathic and conventional dentifrices had no inhibitory effect on *Candida albicans,* whereas herbal dentifrice showed a zone of inhibition at 10 mm at 1:10 dilution (Table 1).

**Table 1:** Zone of inhibition (in mm) of various dentifrices at three different dilutions

Dentifrices	Zone of inhibition (mm)		
	1:5	1:10	1:15
Streptococcus Mutans			
Herbal dentifrice	10	8	6.5
Homeopathic dentifrice	8	7	7
Conventional dentifrice	10	7.5	7
Enterococcus faecalis			
Herbal dentifrice	9	7	-
Homeopathic dentifrice	-	-	-
Conventional dentifrice	9	8	7
Candida albicans			
Herbal dentifrice	-	10	-
Homeopathic dentifrice	-	-	-
Conventional dentifrice	-	-	-

## Discussion

It is well recognized that the oral microbiota possesses a delicate microbial equilibrium and that the illness process begins when this balance is disrupted [3]. *Streptococcus mutans, Enterococcus faecalis*, and *Candida albicans* were selected as test microorganisms for the study because they have been implicated in oral diseases. *Streptococcus mutans* plays a crucial role in the occurrence of dental caries by fermenting carbohydrates, resulting in acid production that results in the demineralization of enamel [6,7].

*Enterococcus faecalis* has not been considered a part of the normal oral microflora but it is regarded as an infectious microorganism associated with failure of endodontic treatments, and it is also found to be present in different layers of the oral biofilm [8]. Furthermore, *Enterococcus faecalis* may facilitate the advancement of periodontal disease [9]. The most frequent opportunistic fungus found isolated from the oral cavity is *Candida albicans*. It is by far the most often identified fungal pathogen from infected root canals, exhibiting resistance to intracanal treatment, and mostly linked to systemic infections as well as mucosal infections (oral candidiasis and denture-related stomatitis) [7,10].

One of the causes of the growth of these bacteria and their detrimental effects is poor dental hygiene. In addition, mechanical plaque management takes time, and some people might not be motivated to do these regularly. Dentifrices now incorporate antibacterial compounds in an attempt to increase the effectiveness of mechanical tooth-cleaning techniques. These include lowering the rate at which plaque accumulates overall, preventing bacteria from adhering to the tooth surface and lowering the number of bacteria in saliva [11,12].

By measuring the zones of inhibition against the investigated pathogens using the agar diffusion method, the antibacterial activity was ascertained. The diffusion phenomenon depends on the physical and chemical properties of tested products such as the diffusion coefficient and the medium where the diffusion occurs.

In our study, herbal and conventional dentifrices at 1:5 dilution showed the highest zone of inhibition of 9 mm, followed by conventional dentifrice at 1:10 dilution with an 8 mm zone of inhibition against *Enterococcus faecalis*. The largest inhibition zone for *Streptococcus mutans* was observed in experiments with conventional and herbal dentifrices of 10 mm at a 1:5 dilution ratio. For *Candida albicans*, the zone of inhibition was found to be higher with herbal dentifrice by about 10 mm at a 1:10 dilution ratio.

The results of this study were on par with the findings of the study done by Basu A et al. which showed that conventional and herbal dentifrice formulations had significant antimicrobial activity than homeopathic dentifrices [13]. Bhat et al. also stated that homeopathy may not be a substitute for good oral hygiene habits and dietary practices. They also emphasized the importance of further research on homeopathic remedies to prove their efficiency [14].

Similarly, the study done by Mohan Kumar et al. showed that herbal toothpaste had similar and slightly better antibacterial activity compared to conventional toothpaste. They also added that herbal toothpaste that had a combination of multiple herbs had better antimicrobial activity compared to those with a single ingredient against certain microorganisms [15].

Careful evaluation and isolation of both aerobes and anaerobes is crucial in the treatment of oro-dental infections since the concept of oral infections has historically been bacteriologically non-specific and has provided no justification for antibacterial treatment [16].

Despite offering valuable insights into the tested dentifrice's antibacterial effectiveness, this study has certain limitations such as the limited number of microorganisms it examined and the in-vitro nature of the tests performed. Also, there has been no investigation of the clinical effects. To stop *Streptococcus mutans *from permanently establishing itself in the oral cavity, it is important to stop its growth during the early stages of colonization. Consequently, reducing caries may be facilitated by brushing the teeth with a dentifrice that exhibits strong antibacterial activity against *S. mutans*. Likewise, immuno-compromised individuals and those who are prone to oral fungal infections like individuals on drug therapy should take a dentifrice with strong inhibitory qualities against *Candida albicans* [17].

## Conclusions

The study results indicate that the conventional and herbal dentifrices were found to be more effective against *Streptococcus mutans *than homeopathic dentifrice used in the study, whereas herbal dentifrice was more effective against* Candida albicans *when compared to conventional and homeopathic dentifrices. In general for managing the oral microbiota, conventional and herbal dentifrices had better anti-microbial activity compared to that of homeopathic dentifrices. So, taking these considerations into account, dentists can recommend dentifrice according to the microorganism causing the specific diseases.
